# Ethical Climate(s), Distributed Leadership, and Work Outcomes: The Mediating Role of Organizational Identification

**DOI:** 10.3389/fpsyg.2020.564112

**Published:** 2021-02-04

**Authors:** Massimiliano Barattucci, Manuel Teresi, Davide Pietroni, Serena Iacobucci, Alessandro Lo Presti, Stefano Pagliaro

**Affiliations:** ^1^Department of Psychology, eCampus University, Novedrate, Italy; ^2^Department of Neurosciences, Imaging and Clinical Sciences, University of Studies G. d’Annunzio Chieti–Pescara, Chieti, Italy; ^3^Department of Psychology, University of Campania “Luigi Vanvitelli”, Caserta, Italy

**Keywords:** ethical climate, distributed leadership, identification, work outcomes, outcomes

## Abstract

Organizational identification (OI) has increasingly attracted scholarly attention as a key factor in understanding organizational processes and in fostering efficient human resource (HR) management. Available evidence shows that organizational ethical climate crucially predicts OI, a key determinant of both employees’ attitudes and behaviors. In the present paper, we examined the relationship between two specific ethical climates (self-interest vs. friendship), distributed leadership (DL), and employees’ attitudes and behaviors, incorporating OI as a core underlying mechanism driving these relationships. Three hundred and forty-two employees filled out questionnaires to examine ethical climate, DL, OI, and a series of measures concerning attitudes and behaviors toward the organization. Structural equation modeling confirmed that a perception of an ethical climate of friendship (but not self-interest) fostered OI, which elicited higher commitment, perceived trust and recommendation, and lower turnover intention. Perception of DL further contributed to increasing OI. Our findings suggest that HR practices should carefully consider employee perceptions of a collectivistic (vs. individualistic) ethical climate, together with perceptions of DL, as key determinants of positive organizational outcomes. We discuss results in light of the social identity approach and present practical implications for HR management.

## Introduction

In the last three decades, both researchers and practitioners interested in organizational processes focused their attention on the psychological link between employees and their organizations. Understanding the strength of such a link is crucial for the development and implementation of efficient human resource (HR) policies and practices. HR management (HRM) must deal with the new operational and organizational scenarios that have recently been unfolding in a timely and creative manner (Cornelissen et al., 2007). As change is no longer a rare event to cope with but rather the norm, HRM is progressively moving its focus toward processes that deal with the communication and sharing of values, visions, and objectives that allow workers to better face these continuous transformations and challenges (He and Brown, 2013; Bednar et al., 2020). Consequently, organizational identification (OI) started playing a key role in strategic management research, primarily for its effects on many motivational factors, work outcomes, attitudes, behavioral intentions, and team dynamics (Meyer et al., 2006; Liu et al., 2011; Smith, 2011; Peters et al., 2013; Zagenczyk et al., 2020).

Recently, the concept of OI has been related to moral characteristics of organizational environment and leadership style. Researchers proposed that employees’ perception of different ethical climates may determine different degrees of OI, and this, in turn, may influence their attitudes and behaviors (Pagliaro et al., 2018; Teresi et al., 2019). In the present paper, building on Pagliaro et al.’s (2018) findings, we explored the role of OI between the perception of ethical climate(s) and employees’ reactions. Moreover, attempting to extend our knowledge about the key role of OI, distributed leadership agency (DLA) (Jønsson et al., 2016) was considered as a further additional antecedent.

### OI, Antecedents, and Outcomes

There has been an increasing interest in applying the social identity approach (Tajfel and Turner, 1979; Turner et al., 1987) to the classical topics of organizational psychology. According to the social identity approach, a fundamental part of people’s identity is derived from the groups they belong to, and this has several relevant consequences in terms of cognition, affect, and behavior.

From the seminal work by Ashfort and Mael (1989), such a theorization has been fruitfully applied to organizational settings. OI refers to the psychological link, coupled with its emotional value, between an employee and his/her organization. Scholars extensively examined organizational behaviors in light of the social identity approach, shedding light on topics such as leader–follower relations, decision making, job strain, turnover intentions, work motivation, and organizational trust (Haslam, 2004). In a recent meta-analysis, Lee et al. (2015) showed a significant effect size between OI and both positive job attitudes and behaviors. Therefore, we anticipated the following:

Hypothesis *1a:* OI will be positively related to positive work outcomes and negatively to turnover intention.

Organizational identification was extensively found to mediate the effects of a wide range of moral organizational and leadership dimensions on many different work outcomes (such as performance, motivational, and behavioral ones) (Islam et al., 2019; Malik et al., 2019; Zappalà et al., 2019; Liu et al., 2020; Miao and Zhou, 2020). Moreover, drawing on the social identity approach and recent indications regarding moral identity, and perceptions about culture, climate, and supervisors’ values, OI can be considered as an antecedent of the interlocking processes of sense-giving and sense-making which help self-categorization as an organizational member (Van Knippenberg and Sleebos, 2006; Hardy and Carlo, 2011; Ellemers and Haslam, 2012). This evidence motivated the examination of OI as a mediator between ethical climate as well as DLA and measured outcomes:

Hypothesis *1b:* *OI will mediate the effects of ethical climate and leadership style on work outcomes*.

### Ethical Work Climate, Moral Norms, and Prescribed Behaviors

Since the late 1980s, broad scientific and managerial debates have developed around the organizational, group, and personal mechanisms involved in the various forms of questionable practices and negative work behaviors that can lead to significant organizational and social costs (e.g., Huang et al., 2017). The concept of ethical climate has started playing a growing role among the seemingly manageable antecedents of employee behavior (Martin and Cullen, 2006; Mayer, 2014; Newman et al., 2017). Ethical work climate has been classically defined as “a set of shared perceptions of procedures and policies, both codified and informal, which shape expectations for ethical behavior within an organization or a company” (Victor and Cullen, 1987). Other scholars proposed alternative definitions focusing their attention on specific organizational aspects rather than on individual aspects, nonetheless confirming ethical climate as a central construct in exploring moral norms and prescribed behaviors at work (e.g., Wang and Hsieh, 2012; Ning and Zhaoyi, 2017). Ethical climate can provide employees with the behavioral guidelines that help them discern what is acceptable from what is sanctionable in the workplace and thus represents a strong group regulation tool (DeRue and Ashford, 2010).

Since Victor and Cullen’s (1987) taxonomy, different types of ethical climates and different ways of differentiating between these have been proposed (e.g., Babin et al., 2000; Schminke et al., 2005). A theoretical distinction can be made between an ethical organizational climate of self-interest (which underlines an individualistic and independent way of dealing with ethical issues within the organization) and an ethical organizational climate of friendship (which instead subsumes a collective and interdependent way of dealing with the same ethical issues; Cullen et al., 1993; Pagliaro et al., 2018; Teresi et al., 2019).

Several studies have examined the impact of (different) ethical climate, acknowledging its practical implications and importance within the organizational life (Newman et al., 2017). Ethical climate has been demonstrated to significantly impact employee’s ethical behavior (e.g., Treviño et al., 1998), attitudes (e.g., Deshpande, 1996; Schwepker, 2001), motivational aspects (commitment, e.g., Babin et al., 2000; proactive customer service performance, e.g., Lau et al., 2017; and helping behavior, e.g., Kalshoven and Boon, 2012), turnover intentions (e.g., Mulki et al., 2006), organizational citizenship behaviors (OCBs) (e.g., Pagliaro et al., 2018), organizational deviance (e.g., Hsieh and Wang, 2016), and a range of counterproductive behaviors, including tardiness or absenteeism (Peterson, 2002; Jaramillo et al., 2006).

In brief, ethical climate is associated with higher positive work behaviors and negatively related to deviant work behaviors (Choi et al., 2015). Specifically, a comparison between an ethical climate promoting benevolent behaviors and one driven by some form of self-interest shows that the former is strongly associated with performance and employees’ positive attitudes and behaviors (e.g., Peterson, 2002; Mayer, 2014). Building on these premises, we anticipate the following:

Hypothesis *2a:* *Friendship ethical climate will be positively related to positive outcomes and negatively to turnover intention*.
Hypothesis *2b:* *Self-interest ethical climate will be negatively related to positive outcomes and positively to turnover intention*.

Recently, Pagliaro et al. (2018); see also Teresi et al., 2019, in a theoretical framework based on the social identity approach, compared the effects of two specific perceived ethical climates on employees’ attitudes and behaviors. They found a positive association between the perception of a friendship climate and OI, which then was positively related to OCB and negatively related to counterproductive work behaviors. Conversely, a negative association between the perception of a self-interest climate and OI emerged. Building on these results, we predicted the following:

Hypothesis *2c:* *Friendship ethical climate will be positively related to OI*.
Hypothesis *2d:* *Self-interest ethical climate will be negatively related to OI*.

### Values, Climate, and Distributed Leadership

Leadership represents one of the fundamental aspects of organizational life, critical in shaping employee attitudes and behavior. While some scholars have focused on the transmission of organizational values from the leader to the employees and its effects on employees’ outcomes (Graber and Kilpatrick, 2008; Mancheno et al., 2009), others examined the associations between styles and types of leadership and ethical climate (Brown et al., 2005; Ning and Zhaoyi, 2017).

Furthermore, empirical evidence highlights that self-managing teams, delegation, and decentralized charts enable companies to better handle change and complexity (Bolden, 2011; Yammarino et al., 2012). Stemming from such evidence, throughout the last 20 years, the concept of cooperative leadership has been attracting a great deal of attention from both management scholars and HRM experts (D’Innocenzo et al., 2014; Wang et al., 2014; Tian et al., 2016). Originally formulated in studies exploring influence processes within groups, distributed leadership (DL) arises when two or more individuals share the roles, responsibilities, and functions of leadership (Jønsson et al., 2016). DL includes a “leader-plus-individuals aspect” (leading should include multiple individuals and focus on the collaboration between leaders and followers) and a “social distribution aspect” (leadership functions are based on the effort of many employees, and tasks are accomplished through interaction between multiple leaders) (Spillane, 2006). This construct is based on common responsibility and initiative (Spillane, 2006); it includes engaging group activity (Bennett et al., 2003) and a pattern of relationships within the norms of the organization (Gronn, 2002). Thus, it is not just a practice carried out *by* multiple individuals, but it is created *with* them.

Based on the concept of DL, DLA represents the degree to which all employees individually experience being actively engaged in leadership activities, managing tasks, and communication at work (Jønsson et al., 2016). Leveraging on the concept of individual as an agent (Mayrowetz, 2008; Gronn, 2009), it denotes how much each employee, with or without formal leadership functions, is involved in leadership tasks, resulting in a positive influence on commitment, satisfaction, and perceived autonomy (Jønsson et al., 2016; Unterrainer et al., 2017; Barattucci et al., 2020). This leads us to hypothesize the following:

Hypothesis *3a:* *DLA will be positively related to positive outcomes and negatively to turnover intention*.

As suggested by Hogg (2001) in his work on leadership through the lens of social identity theory, the contribution of the supervisors allows the representation of organizational values, standards, and norms and reduces in-job and in-role uncertainty of employees, thus facilitating the OI process. Despite the impressive number of studies that confirmed the positive relationship between specific leadership styles (ethical, authentic, transformational, servant, etc.) and OI (e.g., Cheng and Wang, 2015; Van Knippenberg, 2016), no research investigated the relationship between DLA and OI. According to the above-presented background, the present study proposes the following hypothesis:

Hypothesis *3b:* *DLA will be positively related to OI*.

Leadership seems to be fundamental for implementing an ethical climate because whenever leaders demonstrate ethical behavior, their employees will most frequently follow ethical expectations (Dinc and Aydemir, 2014; Newman et al., 2017; Naeem et al., 2019). Literature highlighted that workers’ perception of an employee-oriented ethical climate frequently entails a stronger identification with their company and increases supporting behaviors toward ones’ own organization, extra-role behaviors, efforts for common target, and in-work outcomes (Haslam and Ellemers, 2005; Eisenberger and Stinglhamber, 2011; Teresi et al., 2019).

On those premises, in the present study, we postulate the existence of a mutual interplay of delegation empowerment (perceived as a supportive initiative and based on the collective contribution) and perceptions regarding the organizational ethical identity. Furthermore, we find reasonable the assumption that DLA will positively relate with a friendship ethical climate and not with a self-interest ethical climate and that DLA will relate to many outcomes through OI (Barattucci et al., 2020), and we hypothesized the following:

Hypothesis *4:* *DLA will be positively related to friendship ethical climate and negatively related to self-interest ethical climate*.

### The Present Research

Based on the rationale described above, in the present study, we aimed to examine the role of OI in mediating the effect of two antecedents (perception of ethical climate and DLA) and employees’ attitudes and behavior.

**FIGURE 1:**
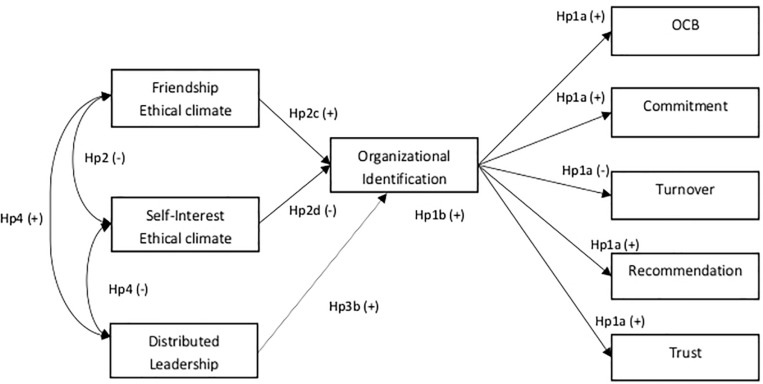
Research model and hypotheses.

Our aim is thus threefold: we first attempt to extend our understanding of the distal consequences of OI, by examining a wide range of work outcomes (trust, commitment, recommendation, OCB, and turnover); the second aim is to replicate Pagliaro et al.’s (2018) findings, showing that an ethical climate of friendship fosters OI, which, in turn, elicits more positive attitudes and behaviors among employees; at the same time, the research set out to show that an ethical climate of self-interest is related in a negative way to OI. Finally, the third aim is to explore whether a specific kind of leadership, namely, DLA, is likely to contribute to enhancing OI. Figure 1 summarizes our empirical model.

A correlational study was designed with employees working in both public and private sectors. The proposed relationships between variables were tested through structural equation modeling (SEM).

## Materials and Methods

### Participants and Procedure

Questionnaires were administered from January to February 2019 in two Italian companies operating in the social services sector: a local office of the Italian National Social Welfare Institution and a private social services company. The total sample was composed of 342 employees (mean age = 48.10, *SD* = 9.54; 189 women, 153 men), 158 in the public company (response rate = 92%), and 184 in the private one (response rate = 93%). The average organizational tenure was 12.13 years (*SD* = 9.50). Management employees represented 18.4% of the sample, white-collar employees about 37.4%, and regular staff about 33.2%. Educational levels were distributed as follows: 28.7% of the workers had a high school degree, 23.1% had a university degree, 9.4% a higher degree, and the remaining completed only compulsory school or hold a professional qualification.

Respondents could decide whether to complete the questionnaire in the paper-and-pencil format or through an online platform. According to their preference, the former received a copy of the questionnaire along with a research presentation and a sealable envelope, and the latter an email with a link to an electronic form. Questionnaires were distributed within organizations by trained researchers. Completed paper-and-pencil questionnaires were put in anonymous envelopes and returned collectively to the researcher after 3 weeks.

### Measures

*Ethical organizational climate of self-interest* was assessed through four items (Cullen et al., 1993; e.g., “In this company, people are mostly out for themselves”; Pagliaro et al., 2018). Responses were given on a 6-point scale (0 = “completely false” to 5 = “completely true”; α = 0.75).

*Ethical organizational climate of friendship* was measured through six items (Cullen et al., 1993; e.g., “In this company, people look out for each other’s good”; Pagliaro et al., 2018) on a scale ranging from 0 (“completely false”) to 5 (“completely true”) (α = 0.76).

*Distributed leadership agency* was assessed with the Italian version (Barattucci et al., 2020) of the DLA scale (Jønsson et al., 2016), in order to evaluate active participation in leadership tasks (α = 0.96). The scale was composed of 11 items, on a 5-point scale (from 0, “completely false,” to 4, “completely true”).

*Organizational identification* was assessed through the Italian adaptation (Manuti and Bosco, 2012) of the original six-item scale by Mael and Ashforth (1992), revised for organizational contexts (e.g., “When someone criticizes my organization, it feels like a personal insult”; α = 0.89; from 0, “completely disagree,” to 5, “completely agree”).

Organizational citizenship behaviors were assessed with the Italian version (Argentero et al., 2008) of the original questionnaire by Podsakoff et al. (1990). The scale includes 15 items (e.g., “Help others who have heavy workloads”; α = 0.89; from 1, “never,” to 7, “always”).

*Commitment* was assessed through 20 items of the Italian form (Pierro et al., 1995) of the commitment scale by Meyer and Allen (1991) (e.g., “I would be very happy to spend the rest of my career with this organization”; α = 0.79; from 0, “completely disagree,” to 5, “completely agree”).

*Perceived organizational trust* was measured with three items (e.g., “I believe that my company is fair”; from 1, “completely disagree,” to 7, “completely agree”α = 0.90), adapted from the international literature (Colquitt and Rodell, 2015).

*Turnover intentions*, or the intention to leave, was assessed through a single item (“If I had the opportunity, I would certainly quit my actual job”; 0 = “strongly disagree” to 6 = “strongly agree”), adapted from the international literature (Waung and Brice, 2007).

*Recommendation*, that is, the overall organizational recommendation, was measured with a single-item assessing the likelihood of encouraging others to apply for a job in the organization, based on Waung and Brice (2007), on a 6-point Likert-scale, ranging from 1 (“strongly disagree”) to 6 (“strongly agree”).

Gender, educational level, and company were included as control variables.

### Data Analysis

This study had a correlational design. All constructs were measured through a single questionnaire, and in order to address response bias and common method variance, we recurred to suggested methods in literature (Podsakoff et al., 2003). Different scale endpoints and formats for the predictor and criterion measures were used in order to reduce method biases caused by commonalities in scale endpoints and anchoring effects.

To test our hypotheses, we conducted correlational and regression analyses with SPSS; SEM analysis with AMOS 22.0 was performed to verify the measurement models and the proposed relationships between variables, using indicators’ covariance matrix and maximum likelihood estimation methods. The following indexes were reported: root mean square error of approximation (RMSEA; acceptable values lower than 0.08; Browne et al., 1993); standardized root mean square residual (SRMR; acceptable values lower than 0.08; Hu and Bentler, 1999); comparative fit index (CFI), for which scores higher than 0.90 are acceptable (Marsh et al., 1996); and normed-fit index (NFI). Furthermore, a bootstrapping procedure (Shrout and Bolger, 2002) was applied to test the mediation effects.

## Results

We conducted a confirmatory factor analysis (CFA) (Anderson and Gerbing, 1988) through AMOS 22.0 to test the construct validity and reliability of the measurement model consisting of the aforementioned scales (ethical climate, DLA, OI, OCB, commitment, and trust). CFA compared different nested models, from a one-factor model to a final one (containing all the included measures), to be confirmed, evaluating intermediate solutions. Four different nested models, from a single-factor model to a model with six factors, were compared based on goodness-of-fit indices (GFIs). From Model A (one factor) to Model D (six factors), results showed an improvement of all indices: Model A (one factor), χ^2^ = 7,226 (df = 651), RMSEA = 0.196, CFI = 0.379, SRMR = 0.239; Model B (two factors), χ^2^ = 6,893 (df = 648), RMSEA = 0.164, CFI = 0.428, SRMR = 0.195; Model C (four factors), χ^2^ = 4,141 (df = 635), RMSEA = 0.109, CFI = 0.835, SRMR = 0.141; and Model D (six factors), χ^2^ = 1,717 (df = 624), *p* < 0.001; RMSEA = 0.072; CFI = 0.946; SRMR = 0.056. The final six-factor model showed acceptable GFIs, overall corroborating a reliable measurement model with items referring to their proper factor.

Table 1 summarizes the descriptive statistics and zero-order correlations for all research variables.

**TABLE 1:** Descriptive statistics and zero-order correlations among the variables of the study.

	*M (SD)*	1	2	3	4	5	6	7	8
1) Friendship E.C.	2.66 (0.99)	–	–	–	–	–	–	–	–
2) Self- Interest E.C.	3.14 (1.11)	−0.423**	–	–	–	–	–	–	–
3)DLA	2.71 (1.11)	336**	0.140*	–	–	–	–	–	–
4) Identification	3.31 (1.13)	0.377**	0.039	0.528**	–	–	–	–	–
5)OCB	5.35 (0.97)	0.338**	−0.011	0.605**	529**	–	–	–	–
6) Commitment	3.01 (0.7)	0.521**	−0.168**	0.401**	0.575**	449**	–	–	–
7) Trust	4.96 (1.51)	0.565**	0.413**	0.261**	0.381**	0.383**	0.486**	–	–
8) Turnover	2.53 (1.69)	−0.338**	0.307**	−0.040	−0.162**	−0.140*	−0.458**	−0.366**	–
9) Recommendation	3.56 (1.48)	0.422**	−0.065	0.332**	0.469**	0.371**	0.564**	346**	−293**

*** p < 0.001, **p < 0.01,*p < 0.05.

Our hypotheses regarding the relationship between the main variables were supported by the correlation analysis. A perceived ethical climate of friendship was negatively correlated with a perceived self-interest ethical climate and turnover intention and positively correlated with DL (Hp 4), OI (Hp 2c), and all the other outcomes (Hp 2a). Self-interest ethical climate was positively correlated with turnover intention and negatively correlated with commitment and trust (Hp 2b partially confirmed). Contrary to our expectations, it was not correlated to OI (Hp 2d not confirmed) or to the other outcomes (organizational recommendation and OCB). DL was positively related to OI and all the other outcomes, except for a negative correlation with turnover intention, thus supporting Hp 3a and 3b. An unexpected low positive correlation emerged between DLA and self-interest ethical climate. OI was positively related to all the outcomes (except for a negative correlation with turnover intention; Hp 1a).

We then performed SEM.

**FIGURE 2:**
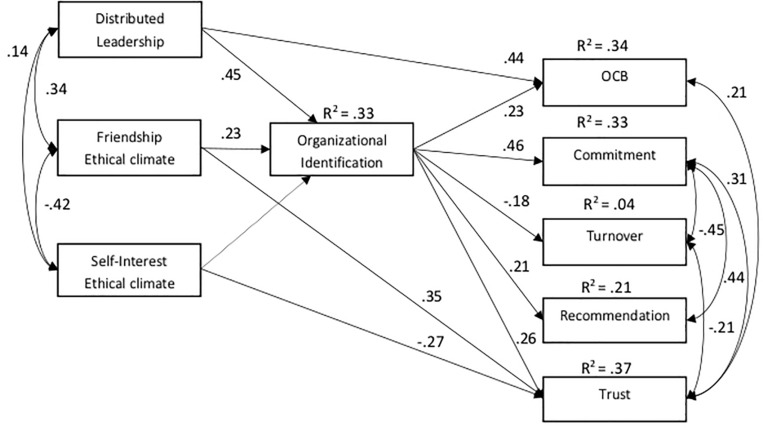
Path analysis of the proposed model.

We specified a model in which both DLA and ethical climate predict OI, which in turn predicts commitment, trust, OCB, organizational recommendation, and turnover. The fit indexes were not completely satisfactory: χ^2^(df = 18) = 140.47, *p* < 0.001; CFI = 0.897, GFI = 0.920; NFI = 0.901; RMSEA = 0.101; SRMR = 0.097. This model was then revised, eliminating the non-significant relationships, and following the results of correlation analysis, direct relationships between DL and OCB and between ethical climate and trust were added (Figure 2 and Table 2). The fit indexes were excellent: χ^2^(df = 18) = 67.5, *p* < 0.001; CFI = 0.956, GFI = 0.961 NFI = 0.952; RMSEA = 0.089; SRMR = 0.080. All of the associations were highly significant (*p* < 0.001).

**TABLE 2:** Standardized path coefficient (regression weights) of the tested model.

			Estimate
Identification	*<* —	Friendship E.C.	0.225
Identification	*< —*	Distributed Leadership	0.453
OCB	*< —*	Distributed Leadership	0.438
Trust	*< —*	Identification	0.258
Trust	*< —*	Self-Interest E.C.	−0.273
Trust	*< —*	Friendship E.C.	0.349
Commitment	*< —*	Identification	0.454
Recommendation	*< —*	Identification	0.206
Turnover	*< —*	Identification	−0.178
OCB	*< —*	Identification	0.224

All the expected associations from OI to the other outcomes were confirmed (OCB: β = 0.23, *p* < 0.001); commitment: β = 0.46, *p* < 0.001; recommendation: β = 0.21, *p* < 0.001; trust: β = 0.26, *p* < 0.001; turnover intention: β = −0.18, *p* < 0.001).

An ethical climate of friendship significantly predicted all the outcomes through OI, except for trust (partial mediation). Results showed that an ethical climate of self-interest did not predict OI, while it directly and negatively predicted trust (β = −0.27, *p* < 0.001).

Distributed leadership agency significantly predicted all outcomes through OI, except for OCB (partial mediation).

Overall, the effect of an ethical climate of friendship and DLA on several outcomes was mediated by OI (Table 3). Thus, a collective and interdependent ethical climate, as well as employees’ perceptions regarding delegation, predicted a stronger OI, which in turn determined better work outcomes (Hp 1b). An ethical climate of friendship, on its own, manages to explain 19% of the variance of OI while, coupled with DL, the proportion of variance explained increases to 33%.

**TABLE 3:** Indirect effects using bootstrapping (2,000 replications) in the tested model.

Indirect effects	Bootstrap		
	B 95%CI [LL, UL]	*SE*	*P*
Friendship Eth. Clim. > Organiz. Ident. > OCB	0.075 [0.043, 0.118]	0.019	0.001
Friendship Eth. Clim. > Organiz. Ident. > Commit.	0.115 [0.071, 0.170]	0.025	0.001
Friendship Eth. Clim. > Organiz. Ident. > Turnover	−0.084 [−0.079, −0.003]	0.021	0.043
Friendship Eth. Clim. > Organiz. Ident. > Recomm.	0.089 [0.053, 0.1461	0.023	0.000
Friendship Eth. Clim. > Organiz. Ident. > Trust	0.064 [0.032, 0.1051	0.019	0.001

	**B 95% CI [LL, UL]**	***SE***	***P***

Individ. Eth. Clim. > Organiz. Ident. > OCB	0.026 [0.002, 0.059]	0.014	0.034
Individ. Eth. Clim. > Organiz. Ident. > Commit.	0.040 [0.001, 0.086]	0.021	0.040
Individ Eth. Clim. > Organiz. Ident. > Turnover	−0.012 [−0.041, 0.000]	0.010	0.080
Individ. Eth. Clim. > Organiz. Ident. > Recomm.	0.031 [0.002, 0.068]	0.017	0.035
Individ. Eth. Clim. > Organiz. Ident. > Trust	0.023 [0.003, 0.051]	0.012	0.027

	**B 95% CI | LL, UL]**	***SE***	***P***

Distr. Leader. Agen. > Organiz. Ident. > OCB	0.114 [0.070, 0.173|	0.026	0.001
Distr. Leader. Agen. > Organiz. Ident. > Commit.	0.175 [0.122, 0.241]	0.031	0.001
Distr. Leader. Agen. > Organiz, Ident. > Turnover	−0.081 [−0.106, 0.007]	0.029	0.044
Distr. Leader. Agen. > Organiz. Ident. > Recomm.	0.136 [0.086, 0.202]	0.030	0.001
Distr. Leader. Agen. > Organiz. Ident. > Trust	0.098 [0.051, 0.160]	0.028	0.001

*All parameter estimates are presented as standardized coefficients. CI, Confidence interval.*

Model invariance across different groups (gender, education, and company) was assessed using a multigroup SEM procedure to estimate chi-square differences between the unconstrained (with original parameters) and the constrained (with equal loading parameters) nested models.

The model shows measurement invariance across private and public social companies, gender, and degree level obtained. Indeed, results did not show any significant difference for company (χ^2^ difference = 4.61, Δdf = 4, *p* > 0.35), gender (χ^2^ difference = 1.71, Δdf = 4, *p* > 0.707), and education (χ^2^ difference = 1.06, Δdf = 8, *p* > 0.788).

## Discussion

How can organizations encourage employees’ positive attitudes and behaviors in the workplace? Which factors foster employees’ identification with and commitment to their organization? In the last decades, the study of organizational processes through the social identity approach has significantly contributed to our knowledge about these relevant questions within both the OI and HRM fields. In the present paper, we connected insights from this approach with the literature concerning two main aspects of organizational life: ethical climate and DL. Extending recent research (Pagliaro et al., 2018; Teresi et al., 2019), we provided evidence about the key role of OI as a mediator of the relationship between perceived ethical climate and employees’ reactions. We found that a perception of a specific ethical climate, based on a collectivistic and interdependent view of organizational life, elicits OI, and this, in turn, induces pro-organizational attitudes and behaviors and discourages turnover intentions. By contrast, when the perceived ethical climate focuses on an individualistic and independent way of approaching organizational processes, employees identify with the organization to a lesser extent. We also provided evidence that a leadership style which strengthens the employees’ perception of being actively engaged in leadership activities, task management, and work communication is also likely to strengthen OI and produce benefits in terms of attitudes and behaviors.

If we refer to social identity theory, identification is generated through socio-cognitive processes of social comparison and categorization, which involve choice-making activities. The worker places himself/herself, the groups of workers, and supervisors in homogeneous social categories, and this process allows him/her to appreciate his/her own characteristics as part of a group or company (Van Dick et al., 2004; Kreiner et al., 2015).

The degree to which organizational values are incorporated through self-conceptualization processes also depends on the managerial initiatives aimed at encouraging the diffusion and the application of these values, which can thus be considered as precursors of OI (Rijswijk et al., 2006; Van Knippenberg and Sleebos, 2006). In this light, our results indicate that when ethical climate and empowerment (e.g., through delegation) are perceived as supportive and based on the collective contribution, workers seem to feel more linked with the organization and to gain positive work outcomes.

### Theoretical Implications

The present research contributes to the development of theoretical knowledge on the role of OI as a mediator between manageable antecedents (climate and leadership) at different levels (individual, group, and organization) and important work outcomes (Soenen and Melkonian, 2016; Wang et al., 2017; Irshad and Bashir, 2020; Nguyen et al., 2020). First, it confirms that environmental, relational, and managerial factors and perceptions (e.g., cultural values, supervisor support, organizational climate, and leadership style) are internalized by workers, thus influencing behaviors and motivational aspects (Stinglhamber et al., 2015; Piccoli et al., 2017; Bednar et al., 2020). Furthermore, this study highlights that both delegation and an ethical climate of friendship support OI processes, with a positive impact on work outcomes. On the other hand, the perception of an ethical climate of self-interest does not seem to contribute to the OI process. Overall, our results are in line with previous studies that show that employee’s perceptions of supportive organizational climates and practices (characterized by ethics and responsibility, the sharing of values, the attention to the health and safety of workers, and morality) have effects on important work outcomes through OI. Moreover, in line with these results, this study shows that perceptions regarding empowerment and delegation (conceptualized as DLA) have a significant positive relation with an ethical climate of friendship, a significant negative relation with a self-interest ethical climate, and a significant positive effect on OI (Unterrainer et al., 2017; Barattucci et al., 2020). To the best of our knowledge, this is the first study suggesting that DLA has a positive impact on work outcomes through OI, thus opening up new possible theoretical debates and directions.

### Practical Implications

The present research can offer many insights for practitioners. First of all, HRM should monitor the evolution of employees’ identification with their organization through a continuous and dynamic process of sense-making (Ashforth and Schinoff, 2016), so as to avoid “snap-shot” approaches (Bednar et al., 2020). Also, this process should incorporate operational practices specifically aimed at positively impacting the OI: interventions on resources (autonomy, training, jobs, responsibilities, information, etc.), managerial support, communication, and work climate (Eisenberger and Stinglhamber, 2011). Results also highlight the importance of the creation of an effective work climate based on cooperation, support, friendliness, and delegation, in order to promote adequate OI and act positively on outcomes such as turnover, satisfaction, and trust (Stinglhamber et al., 2015).

Moreover, from a practical point of view, it confirms that different ethical climates are likely to impact differently on employees’ reactions – for example, in terms of commitment, OCB, perceived organizational morality, and turnover intention. If not properly addressed, unethical individual and/or group behaviors could lead to the perception that such conduct is acceptable, potentially paving the way for further issues like absenteeism, turnover, tardiness, social loafing, low satisfaction, and low commitment. On the contrary, organizations capable of keeping ethical standards of behavior and fostering a clear ethical perception among their employees could potentially benefit from a positive array of likewise moral behaviors.

A further practical implication that may be derived from the present findings is related to the efficacy of DL in fostering employees’ identification with, and commitment to, the organization. We provided evidence about the fact that a leadership style that actively involves employees in leadership activities and managing tasks is likely to create a *we-ness* (Ashfort et al., 2011), reflected in OI, that further improves positive reactions. Thus, companies may be particularly interested in eliciting such a leadership style, and this may influence the training and development plans of the organization.

### Limitations and Future Directions

The study is not exempt from some limitations that are worth noting and that could be considered in future developments. First of all, the nature of the data is cross-sectional. Future studies may be directed at disentangling the causal direction we hypothesized and tested here, although some indications coming from findings of prior simulation studies conducted in a laboratory setting reassure us about the validity of our assumptions (Teresi et al., 2019). Moreover, we are further reassured by prior literature predicting the role of OI on employees’ reactions. It seems reasonable to expect that this could be more of a recursive relationship, in which identification fosters positive attitudes and behaviors, and in turn, endorsing a positive view of the company further improves identification.

Another limitation is the absence of a (negative) relationship between self-interest climate and identification, as emerged in Pagliaro et al.’s (2018) study. Even though this calls for further attention in future studies, the present paper focused more on the positive relationship between friendship, ethical climate, and identification, and in this sense, our findings are in line with previous research. It is also worth noting that a self-interest ethical climate was directly and negatively related to organizational trust, thus providing further argument supporting our general hypothesis.

In order to overcome the limitations inherent to self-administered questionnaires, future research should consider implementing third-part evaluations by supervisors or colleagues as well as objective data and possibly measurements of variables at the group level. Moreover, it would be advisable to go beyond the correlational design and evaluate longitudinal, cross-lagged, or experimental design.

Overall, the present research confirms that understanding the dynamics of OI is crucial in order to manage personnel attitudes and behaviors.

## Data Availability Statement

The raw data supporting the conclusions of this article will be made available by the authors, without undue reservation.

## Ethics Statement

The studies involving human participants were reviewed and approved by the Comitato Etico dell’Università eCampus. The patients/participants provided their written informed consent to participate in this study.

## Author Contributions

SP, MB, and AL: conceptualization and methodology. MB, MT, DP, SI, and SP: formal analysis. MT, SP, and MB: investigation and writing – original draft preparation. MB and AL: data curation. DP, SI, AL, and SP: writing – review and editing. All authors contributed to the article and approved the submitted version.

## Conflict of Interest

The authors declare that the research was conducted in the absence of any commercial or financial relationships that could be construed as a potential conflict of interest.
